# Transition metal homoeostasis is key to metabolism and drug tolerance of *Mycobacterium abscessus*

**DOI:** 10.1038/s44259-024-00042-7

**Published:** 2024-09-30

**Authors:** Yi Liu, Katy Murphy, Nadia Fernandes, Rebekah E. T. Moore, Ivana Pennisi, Richard Williams, Mark Rehkämper, Gerald Larrouy-Maumus

**Affiliations:** 1 Centre for Bacterial Resistance Biology, Department of Life Sciences, Faculty of Natural Sciences, Imperial College London, London, UK; 2 Department of Earth Science and Engineering, Royal School of Mines, Imperial College London, London, UK; 3 Imperial BRC Genomics Faculty, Imperial College London, London, UK

**Keywords:** Antibacterial drug resistance, Antimicrobials

## Abstract

Antimicrobial resistance (AMR) is one of the major challenges humans are facing this century. Understanding the mechanisms behind the rise of AMR is therefore crucial to tackling this global threat. The presence of transition metals is one of the growth-limiting factors for both environmental and pathogenic bacteria, and the mechanisms that bacteria use to adapt to and survive under transition metal toxicity resemble those correlated with the rise of AMR. A deeper understanding of transition metal toxicity and its potential as an antimicrobial agent will expand our knowledge of AMR and assist the development of therapeutic strategies. In this study, we investigate the antimicrobial effect of two transition metal ions, namely cobalt (Co^2+^) and nickel (Ni^2+^), on the non-tuberculous environmental mycobacterium and the opportunistic human pathogen *Mycobacterium abscessus*. The minimum inhibitory concentrations of Co^2+^ and Ni^2+^ on *M. abscessus* were first quantified and their impact on the bacterial intracellular metallome was investigated. A multi-omics strategy that combines transcriptomics, bioenergetics, metabolomics, and phenotypic assays was designed to further investigate the mechanisms behind the effects of transition metals. We show that transition metals induced growth defect and changes in transcriptome and carbon metabolism in *M. abscessus*, while the induction of the glyoxylate shunt and the WhiB7 regulon in response to metal stresses could be the key response that led to higher AMR levels. Meanwhile, transition metal treatment alters the bacterial response to clinically relevant antibiotics and enhances the uptake of clarithromycin into bacterial cells, leading to increased efficacy. This work provides insights into the tolerance mechanisms of *M. abscessus* to transition metal toxicity and demonstrates the possibility of using transition metals to adjuvant the efficacy of currently using antimicrobials against *M. abscessus* infections.

## Introduction

Antimicrobial resistance (AMR) has been recognised as one of the greatest threats that the world is currently facing, leading to more than 30,000 directly attributed deaths annually and substantial economic impact^1–3^. Coordinated multidisciplinary actions and deeper understanding of the causes and mechanisms of AMR are required to tackle this multifaceted global problem.

Transition metal ions are ubiquitous in the environment, and although only required by organisms in trace amounts, they play pivotal roles in catalytic, structural, and regulatory aspects of the biological system^4,5^. Meanwhile, metal toxicity arises with excessive transition metal intake, affecting various parts of physiology, including enzyme function, transcription, cellular redox balance, and overall metal homoeostasis^6–8^. This underlines the historical use of transition metals, such as copper and silver, as antimicrobial agents^9,10^. Meanwhile, bacteria have evolved strategies to circumvent metal toxicity during colonisation in different environments. These strategies include metal sensing and regulatory proteins, expression of metal efflux systems, and production of sequestering proteins^5,11,12^. This is a common phenomenon for environmental bacteria, which thrive in various environments with the accumulation of metal ions, such as cobalt (Co^2+^) and nickel (Ni^2+^), with concentrations of up to mM levels^13–16^.

Investigating the intriguing interplay between transition metal toxicity and bacterial metal resistance could provide valuable cues for further exploitation of the potential of transition metals as novel antimicrobials. Furthermore, metal resistance mechanisms are conceptually similar to some AMR mechanisms, raising the possibility that AMR could arise as a by-product of the former^17,18^. For example, as the major form of metal resistance, active ion export by efflux pumps has been linked with higher AMR in *Escherichia coli*, *Salmonella enterica,* and *Pseudomonas aeruginosa*^19–22^. It has also been shown that transition metal stress accentuates the rise and spread of AMR by acting as reservoirs of AMR genes, which may be transferred from environmental bacteria to pathogenic species^23^. For example, the use of copper and zinc compounds in agricultural fields and the release of such compounds into wastewater were found to be correlated to a rise of metal-tolerant bacteria with a higher incidence of AMR^15,16,24,25^. Long-term exposure to Ni^2+^ contamination was also reported to increase the frequency of AMR genes in agricultural fields^26^. These studies clearly reveal correlations between transition metal toxicity and the rise of AMR through similar bacterial responses, although our understanding of this relationship and the multifaceted roles of transition metals in bacterial survival needs to be further expanded.

Here we report on the antimicrobial effect and mechanisms of the transition metal ions Co^2+^ and Ni^2+^ on the environmental bacteria *Mycobacterium abscessus*. The typical natural habitats of *M. abscessus* include soil and natural water system, but it can also accumulate in household water circuits, including in humidifiers and showerheads^27–29^. As an opportunistic human pathogen and a “clinical nightmare”, *M. abscessus* has various properties that contribute to its high resistance and tolerance to antibiotics^30^. As *M. abscessus* is highly resistant to many antimycobacterial antibiotics, such as the frontline antibiotics rifampicin and streptomycin, it is difficult to set up effective and safe treatment regimens for infections^31^. Investigating the potential of using transition metals as antimicrobials, either alone or in combination with other currently used antibiotics, could expand a new strategy to treat this highly resistant bacterium and other pathogenic species.

In this study, a multi-pronged approach, encompassing transcriptomics, metallomics, metabolomics, bioenergetics, and phenotypic analysis, was employed to map the global response of *M. abscessus* to transition metal stress and its link to AMR levels.

## Results

### Transition metal treatments led to growth defects and remodelling of the intracellular metallome of *M. abscessus*

**Fig. 1:**
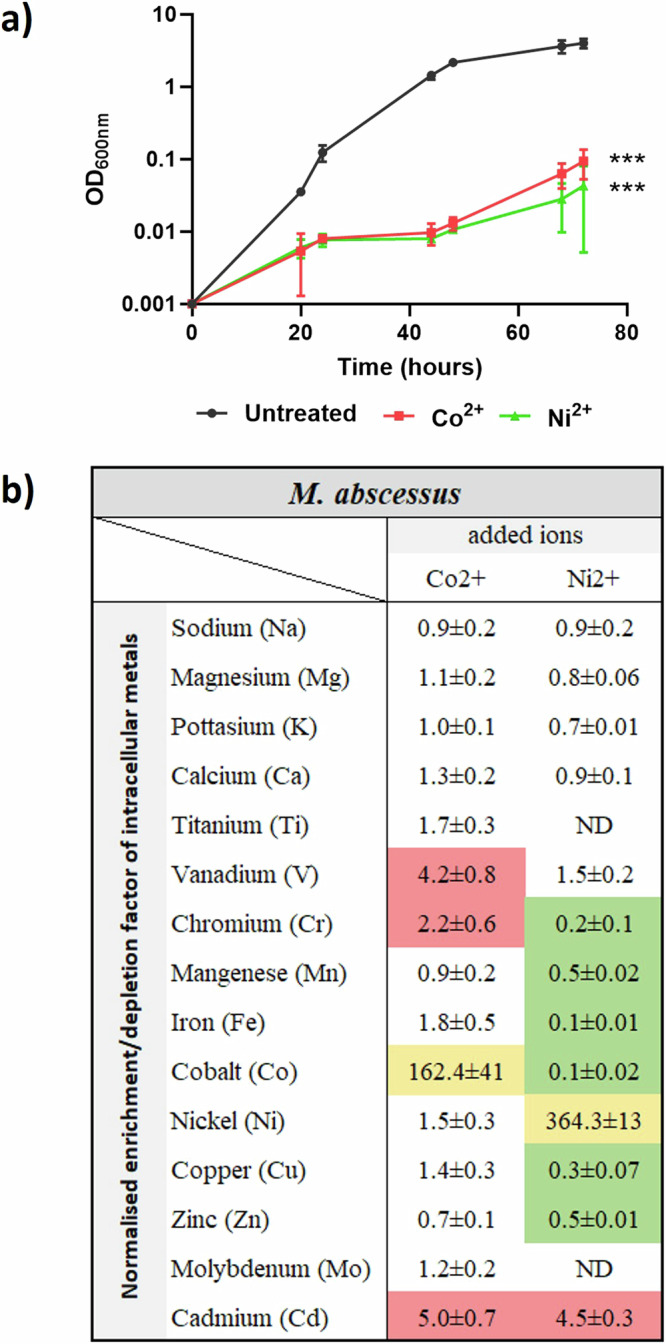
Transition metal treatment changes *M. abscessus* growth profile and intracellular metal homoeostasis. **a** Growth curve of *M. abscessus* untreated (grey) or treated with sub-MIC Co^2+^ (red) or Ni^2+^ (green). Statistical significance levels between ion-treatment groups and the untreated group at *t* = 44 h and *t* = 72 h were calculated by unpaired *t* tests and are *p* < 0.001. **b** Changes in intracellular metal concentrations in *M. abscessus* after treatment with Co^2+^ or Ni^2+^. Data are normalised with viable cell counts and to the results obtained for control experiments with bacteria not treated with transition metals. The data shown are hence expressed as enrichment or depletion factors. Changes in the corresponding ion for each condition are labelled yellow. Significant changes for other ions are labelled in red (enrichment factor greater than 2) or green (depletion factor <0.5). ND no valid data for the element. For all experiments, data are averaged from at least three independent biological replicates and expressed as mean ± standard deviation.

The minimal inhibitory concentrations (MICs) were first quantified by resazurin microtiter assays (REMA). MIC_50_ was defined as the lowest concentration of the corresponding transition metal ion that resulted in the non-replication or killing of at least half of the bacterial population, as read from the colour development of resazurin. The sub-MIC_50_ concentrations, which correspond to 50% of the MIC values, were used in subsequent studies as a basal stress condition for *M. abscessus*. The sub-MIC_50_ was 625 µM for Co^2+^ and 2.5 mM for Ni^2+^. To determine the impact of these concentrations on the viability of *M. abscessus*, growth curves were determined (Fig. 1a). Treatment with sub-MIC_50_ concentrations of Co^2+^ or Ni^2+^ led to significant growth defects after 72-hour incubation compared to untreated bacteria (*p* = 0.0004 and 0.0003, respectively). The dose-dependent effect of the two transition metal ions was also investigated with concentration gradients of each ion (Supplementary Fig. 1), showing that the use of sub-MIC_50_ concentrations led to moderate growth defects on *M. abscessus*.

To study changes in the intracellular metallome of *M. abscessus* that were induced by transition metal ion treatments, metallomics studies were performed using inductively coupled plasma mass spectrometry (ICP-MS). To better present the changes in intracellular ion concentrations, whilst accounting for variations in the numbers of bacteria present in samples, the measured data were normalised to colony-forming units (CFU). The intracellular transition metal concentrations measured for untreated bacteria were furthermore used as controls to account for the presence of extraneous ions. The data are then presented as enrichment or depletion factors relative to non-ion-treated bacteria (Fig. 1b). Treatment with metal ions induced intracellular accumulations of the corresponding ions in each group. The Co^2+^ treatment was able to increase the intracellular concentrations of vanadium, chromium, and cadmium by factors of 4.2, 2.2, and 5.0, respectively, but did not affect the homoeostasis of other transition metals. Meanwhile, the Ni^2+^ treatment induced a 2 to 10-fold reduction in the concentrations of chromium, manganese, iron, cobalt, copper, zinc, and cadmium (Fig. 1b). It is of note that the enrichment factors for cadmium are similar for both treatments, at ~4.5–5, suggesting that the divalent ions tested here both influence cadmium levels in *M. abscessus* in a similar, non-specific way. Non-transition metals, including sodium, magnesium, potassium, and calcium, were not affected by any transition metal treatment. Taken together, these data show that Ni^2+^ was able to induce greater changes in the intracellular metallome compared to Co^2+^.

### Transition metal treatments altered the transcriptome of *M. abscessus*

**Fig. 2:**
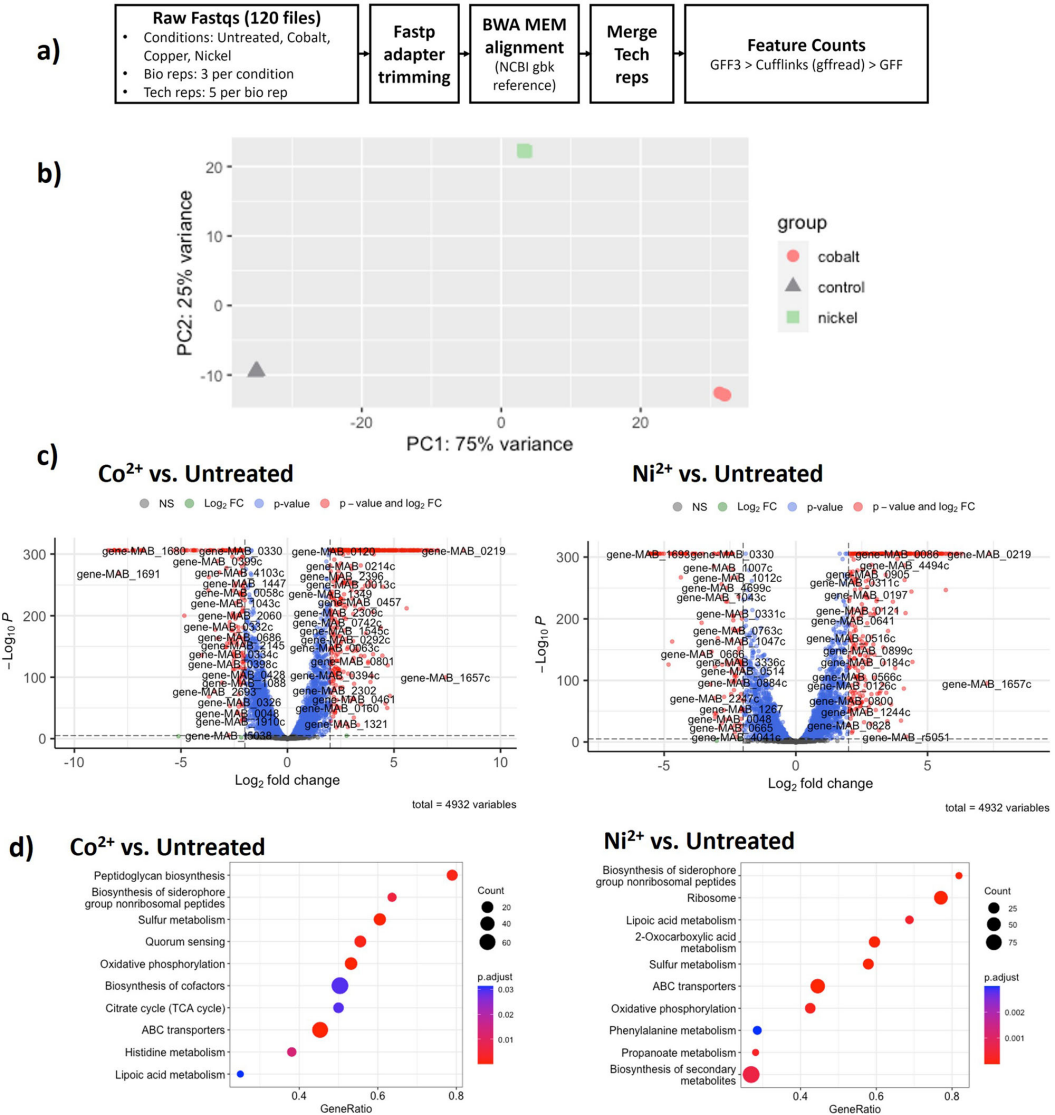
RNA sequencing data for *M. abscessus* treated with transition metal ions. **a** Schematic workflow of RNA sequencing data extraction, quality control and data analysis used in this study. **b** PCA plot showing the distribution for three bacterial conditions, each with three biological replicates. **c** Volcano plots showing the distribution of genes for each ion-treatment group compared with the untreated control, filtered with cutoff fold change (log_2_FC > 2) and levels of significance (*p* < 0.05). **d** Gene set enrichment analysis (GSEA) for Co^2+^- and Ni^2+^-treated *M. abscessus* cultures compared with untreated bacteria. The relative size of each dot on the graph shows the number of differently regulated genes in each pathway, and the colour indicates the level of significance. The relative position on the × axis (gene ratio) denotes the proportion of differently expressed genes in a given pathway, in the range of 0–1.

Given the roles that transition metal ions play in biological systems as cofactors and modulators, it is likely that treatment with transition metal ions could alter the transcriptome of bacteria. To obtain an overview of such effects, RNA sequencing was performed on untreated and metal ion-treated *M. abscessus* (Fig. 2a; Supplementary Data 1–2). By performing the principal component analysis (PCA), the differences between treatment groups can be visualised, with the replicates within each group clustered and separated from other groups (Fig. 2b). The volcano plots (Fig. 2c) also provide an overview of significant changes (log_2_FC > 2, *p* < 0.05) for the genes of each ion-treated group. Gene set enrichment analysis (GSEA) was used to analyse the related pathways with changes at the transcriptomic level in each ion group, in comparison to the untreated control (Fig. 2d). Pathways with significant global changes in gene expression levels were identified for the Co^2+^ and Ni^2+^ groups. In detail, changes were observed for the sulphur metabolism, siderophore biosynthesis, ABC transporters, central carbon metabolism, and biosynthesis of several amino acids.

The central carbon metabolism, including the tricarboxylic acid (TCA) cycle, was predicted to be affected by the Co^2+^ and Ni^2+^ treatments, based on the growth profiles presented previously (Fig. 1a). In the Ni^2+^-treated group, overall upregulation of the genes of the pyruvate dehydrogenase complex was identified with log_2_FC between 0.60 and 4.47, while there was no significant change in the Co^2+^ group. The aconitate hydratase gene MAB_2730 (log_2_FC = 1.58, *p* < 0.0001) and the 2-oxoglutarate dehydrogenase gene *sucA* (MAB_1393c, log_2_FC = 1.02, *p* < 0.0001) also showed significant upregulation in the Ni^2+^ group, suggesting increased activity of the oxidative steps of the TCA cycle. In contrast, enzymes in the reductive steps of the TCA cycle that convert the substrates back into oxaloacetate, including succinyl-CoA synthetase (MAB_1056-1057, log_2_FC = −1.59 and −1.34, *p* < 0.0001), succinate dehydrogenase (MAB_3673-3674 and MAB_4422-4423, log_2_FC between −1.31 and −1.84, *p* < 0.0001) and fumarate hydratase (MAB_1250c, log_2_FC = −1.05, *p* < 0.0001), were downregulated after Co^2+^ treatment. Treatment with Ni^2+^ did not generate such significant enzymatic changes except for two succinate dehydrogenase subunits MAB_4422 (log_2_FC = −1.91, *p* < 0.0001) and MAB_4423 (log_2_FC = −1.80, *p* < 0.0001). Since succinate dehydrogenase is a component of the electron transport chain (ETC), the downregulation of this enzyme complex may affect not only the TCA cycle but also the oxidative phosphorylation (OXPHOS) further downstream. Lastly, the isocitrate lyase *aceA* (MAB_4095c), which is responsible for converting isocitrate into succinate and malate through the intermediate glyoxylate, was found to be upregulated in both the Co^2+^ and Ni^2+^ groups (log_2_FC = 1.62 and 2.50, respectively, both with *p* < 0.0001), suggesting activation of the glyoxylate shunt (Supplementary Data 3).

Changes in TCA cycle enzyme activities may affect the activity of glycolytic pathways, and it has been reported that bacteria such as *M. tuberculosis* alter the activities of these pathways to survive under unfavourable conditions^32,33^. In the Co^2+^ group, genes such as the fructose bisphosphate (FBP) aldolase MAB_4254c and the enolase MAB_1165 were significantly downregulated (log_2_FC = −1.25 and −1.05 respectively, both with *p* < 0.0001). Similar significant effects were found after Ni^2+^ treatment (MAB_4254c: log_2_FC = -0.73; MAB_1165: log_2_FC = -0.34, both with *p* < 0.0001). In the Ni^2+^ group, there were only nonsignificant changes in the pathway upstream of phosphoenolpyruvate, but the conversion between phosphoenolpyruvate, pyruvate, and acetyl-CoA, involving pyruvate kinases and pyruvate dehydrogenases, was upregulated (Supplementary Data 4).

Biosynthesis of amino acids is related to the central carbon metabolism and these pathways were also characterised by GSEA. After treatment with Ni^2+^, expression level changes were found in more than 40% of relevant genes, including the serine-glycine-threonine and cysteine-methionine pathways (Supplementary Data 5). This suggests that the Ni^2+^ treatment was associated with an altered metabolism of amino acids as a consequence of a remodelled central carbon metabolism.

The expression levels of genes belonging to OXPHOS were also investigated, as GSEA indicated an overall downregulation of this pathway (Supplementary Data 6). In addition to succinate dehydrogenase (complex II of the ETC), downregulation of the genes in the *nuo* operon, which encodes the NADH dehydrogenase (NADH-quinone oxidoreductase) subunits, was observed for both the Co^2+^ and Ni^2+^ groups. An additional probable NADH dehydrogenase gene, MAB_2429c, was found to be upregulated (log_2_FC = 1.13 and 1.38 in Co^2+^ and Ni^2+^ groups, respectively, both with *p* < 0.0001), and this could be the alternative electron donor for the bacteria. In both groups, downregulation of the ATP synthase (F-type ATPase) subunits was found (log_2_FC between −2.00 and −2.85 in the Co^2+^ group and between −1.04 and −1.72 in the Ni^2+^ group, all with *p* < 0.0001). For the Co^2+^ group, additional genes that encode subunits of the cytochrome c oxidoreductase and the cytochrome bd complex were downregulated with log_2_FC = −1.62 and −2.50, respectively (both with *p* < 0.0001). These results suggest that OXPHOS might be impaired in Co^2+^- and Ni^2+^-treated bacteria.

Finally, the expression levels of *whiB7* and its regulating genes were investigated based on the reported WhiB7 regulon^34^. After treatment with either Co^2+^ or Ni^2+^, upregulation of *whiB7* was found with log_2_FC of 4.59 (Co^2+^) and 2.04 (Ni^2+^), respectively (both with *p* < 0.0001; Supplementary Data 7). A general upregulation of the WhiB7 regulon was hence observed. Despite a significant proportion of unannotated genes, a large number of genes may be related to molecule transport through the cell envelope, including transporters and efflux pumps from different structural families. For example, the two putative ABC transporters MAB_2355c and MAB_1846 were reported to correlate with resistance to amikacin and clarithromycin^35^, and both were found to be significantly upregulated in the Co^2+^ group (Log_2_FC = 3.35 and 2.03, respectively, both with *p* < 0.0001) and the Ni^2+^ group (log_2_FC = 2.40 and 1.63, respectively, both with *p* < 0.0001). Upregulation of several known AMR genes, such as the macrolide resistance gene *erm*(41) (MAB_2297) and the aminoglycoside resistance gene *eis2* (MAB_4532c), could also be associated with changes in resistance and tolerance levels to these antibiotics.

To summarise, these data show that on exposure to sub-MIC_50_ concentrations of Co^2+^ and Ni^2+^, *M. abscessus* remodels its transcriptome, by inducing changes in the expression of the known transcriptional regulator *whiB7* and by altering the expression of genes that are part of the central carbon metabolism.

### Remodelled carbon metabolism of *M. abscessus* in response to transition metal ion treatments

**Fig. 3:**
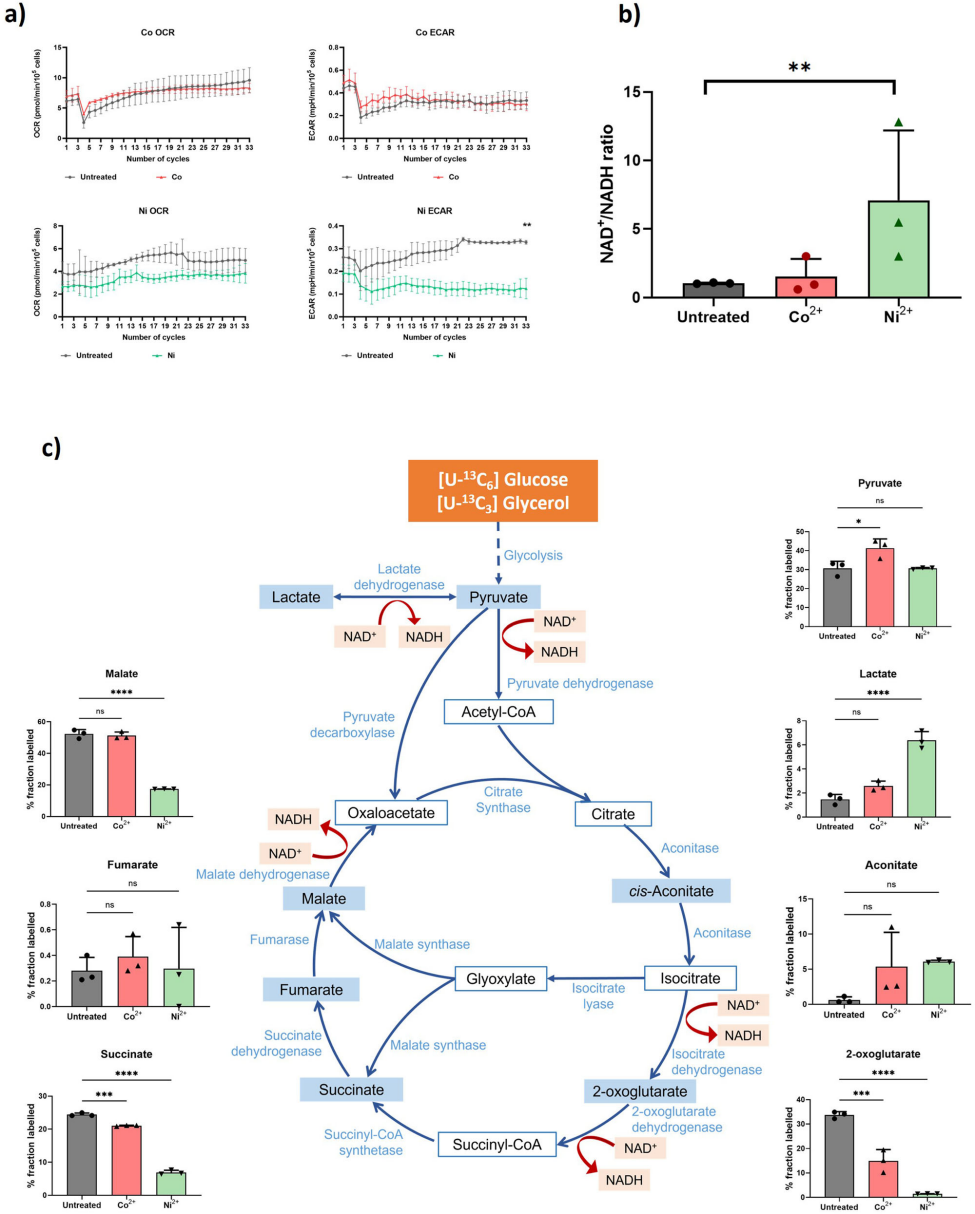
Transition metal treatment leads to changes in the bioenergetics and metabolome of *M. abscessus.* **a** Kinetic quantification of the oxygen consumption rate (OCR, left) and extracellular acidification rate (ECAR, right) in the presence of metal ions (coloured) compared with the untreated controls (grey). Each panel is representative of three independent biological replicates. Statistical significance was measured by unpaired *t* tests as *p* < 0.01. **b** NAD^+^/NADH ratios of transition metal-treated bacteria. The ratios are normalised to the untreated group. Data are averaged from three biological replicates with individual results shown as dots on each bar, and the statistical significance of *p* < 0.01 was measured by one-way ANOVA. **c** Schematic graph of the TCA cycle with key metabolites and enzymes involved. Involvement of the NAD^+^/NADH conversion is shown by red arrows. ^13^C incorporation was measured for metabolites in the pathway. Each panel shows results obtained for three independent biological replicates. Statistical significance was measured by one-way ANOVA with *p < 0.05, **p < 0.01, ***p < 0.001, ****p < 0.0001, ns: not significant.

The bioenergetic and metabolic states of bacteria are interrelated to growth and have been shown to affect bacterial responses to various stresses and antibiotic efficacy^36^. To further investigate the changes in carbon metabolism following transition metal treatments, bioenergetic studies were performed using a Seahorse XFp flux analyser, which allowed real-time measurements of the bioenergetic state of live cells^37^. During the assay, two rates were simultaneously measured: the oxygen consumption rate (OCR) and the extracellular acidification rate (ECAR). These were monitored for 33 cycles, with 6-minute intervals between each cycle, as readouts of respiration and the central carbon metabolism, respectively, using unbuffered culture media^38^ (Fig. 3a).

For all groups and conditions, the OCR showed a steady increase over time after injection of either water or transition metal solutions, and no significant differences were observed between conditions. Meanwhile, the ECAR of bacteria after Ni^2+^ treatment decreased and remained at a lower level compared to the untreated bacteria throughout the assay, with significant differences from cycle 9 (*p* = 0.0377) until the end of the assay (*p* = 0.0014). This suggests shutdown of carbon catabolic pathways specifically due to supplementation of Ni^2+^ but not Co^2+^.

ECAR is an indicator of glycolysis and TCA cycle activity. During anaerobic glycolysis, formation of lactate and H^+^ from glucose is the source of glycolytic acidification, while the release of CO_2_ from the TCA cycle, during conversion of isocitrate to 2-oxoglutarate and subsequently succinyl-CoA, contributes to respiratory acidification^39^. Imbalance of the NAD^+^/NADH cofactor pair has been used as an indicator of altered carbon catabolism due to its wide involvement in these pathways^40^. Here, the concentrations of NAD^+^ and NADH were measured and the NAD^+^/NADH ratio determined for each condition was employed to validate the findings of the Seahorse analyses (Fig. 3b). While there were no significant change after the Co^2+^ treatment, the Ni^2+^ treatment led to a 12-fold higher ratio of NAD^+^/NADH. This result, consistent with the Seahorse analysis, suggests altered activities of glycolysis and the TCA cycle in Ni^2+^-treated bacteria.

To visualise the changes in central carbon metabolism and its related pathways at the metabolomic scale, incorporation of a stable isotope tracer into these pathways was monitored using [^13^C_3_-glycerol] and [^13^C_6_-glucose]. These carbon sources were fed into glycolysis and subsequently the TCA cycle, which were monitored by measuring the label incorporation in the metabolites (Fig. 3c).

Ni^2+^ treatment did not alter the total ^13^C incorporation into pyruvate, while the Co^2+^ treatment group showed a 25% increase in total ^13^C incorporation (*p* = 0.0395). These results may indicate that, even though the transcriptomics data revealed a downregulation of the glycolysis pathway, the overall activity of the pathway remained stable. There was a significantly higher incorporation of ^13^C into lactate after the Ni^2+^ treatment, indicating an increased flow of carbon from pyruvate to lactate in this group. The latter observation may be related to the roles of the lactate dehydrogenase in catalysing the interconversion of pyruvate and lactate, in a bacterial response to reactive oxygen species (ROS) stress caused by the transition metal supplementation.

Among the TCA cycle metabolites, the turnover of 2-oxoglutarate was decreased in both ion-treated groups, from 33% in untreated bacteria down to 12% (Co^2+^ treatments, *p* < 0.001) and 1% (Ni^2+^ group, *p* < 0.0001), suggesting reduction of 2-oxoglutarate biosynthesis. The effect of Ni^2+^ on the overall activity of the TCA cycle was more profound than that of Co^2+^, with a significantly lower turnover of succinate and malate. The decreased activity of the reductive half of the TCA cycle may be linked to the lower ECAR and the NAD^+^/NADH imbalance that were observed in bioenergetic studies.

Taken together, treatments with transition metal ions altered bacterial bioenergetics and metabolomics. This is clearly shown by the reduced turnover of metabolites that are part of the central carbon metabolism and this may be linked to changes in bioenergetic states.

### Ni^2+^ worked synergistically with itaconate and prevented regrowth of *M. abscessus*

The metabolomic and bioenergetic analyses show that Ni^2+^ supplementation alters the central carbon metabolism profoundly, whilst RNA-seq reveal changes in the expression of the glyoxylate shunt. As indicated by the drastic reduction of carbon turnover in 2-oxoglutarate and the subsequent restoration in downstream TCA cycle metabolites, *M. abscessus* may utilise the glyoxylate shunt to bypass the possible blockage of the TCA cycle and maintain its carbon metabolism (Fig. 3c). Considering the important role of the glyoxylate shunt in bacterial physiology, inhibition of this pathway may result in bacterial killing, and transition metals may work synergistically with the known inhibitors of this pathway. To test this hypothesis, *M. abscessus* was treated with Ni^2+^ in combination with itaconate, which is an immunomodulatory metabolite produced by the human immune system and a known inhibitor of isocitrate lyase (ICL), the first enzyme involved in the glyoxylate shunt^41^. The MIC_50_ of itaconate to *M. abscessus* was first tested by REMA, then the effect of Ni^2+^ supplementation on the MIC was tested. There was no difference between the MIC_50_ of itaconate to untreated and Ni^2+^-treated bacteria (6.25 mM).

**Fig. 4:**
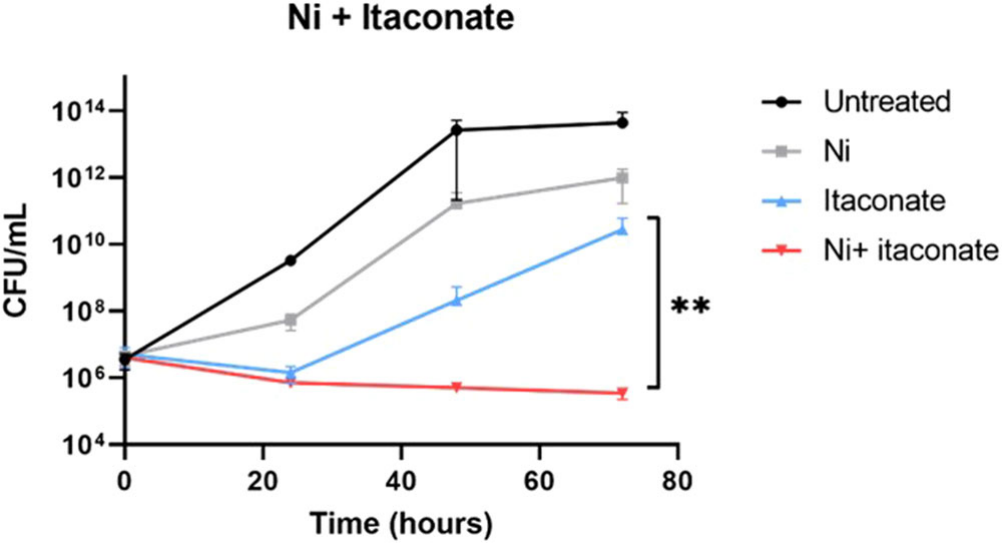
Time-killing curve for *M. abscessus* treated with Ni^2+^ and itaconate. Results are averaged from two biological replicates, each with two technical replicates. The significance level of the difference between the itaconate (blue) and Ni plus itaconate (red) groups was evaluated by unpaired *t* tests (*p* < 0.01).

The killing kinetics of the Ni^2+^-itaconate combination was also tested to further investigate the effect of Ni^2+^ on the efficacy of itaconate (Fig. 4). Itaconate treatment led to bacterial growth arrest for 24 hours and a biphasic regrowing pattern after 24 hours. At the end of the assay, there was a significant growth defect in the Ni^2+^ group compared with the untreated group (*p* = 0.0087). Meanwhile, the combination of Ni^2+^ and itaconate was able to inhibit the regrowth pattern of the bacterial culture, resulting in a static growth curve that differed significantly (*p* = 0.0058) from the itaconate-only group.

Taken together, these data consolidate the observation that the Ni^2+^ treatment of *M. abscessus* was associated with an altered expression of genes that are part of glyoxylate shunt, to bypass inhibition of the oxidative branch of the TCA cycle, as evidenced by the stable isotope tracing experiment.

### Transition metal ion altered bacterial response to antibiotics

**Fig. 5:**
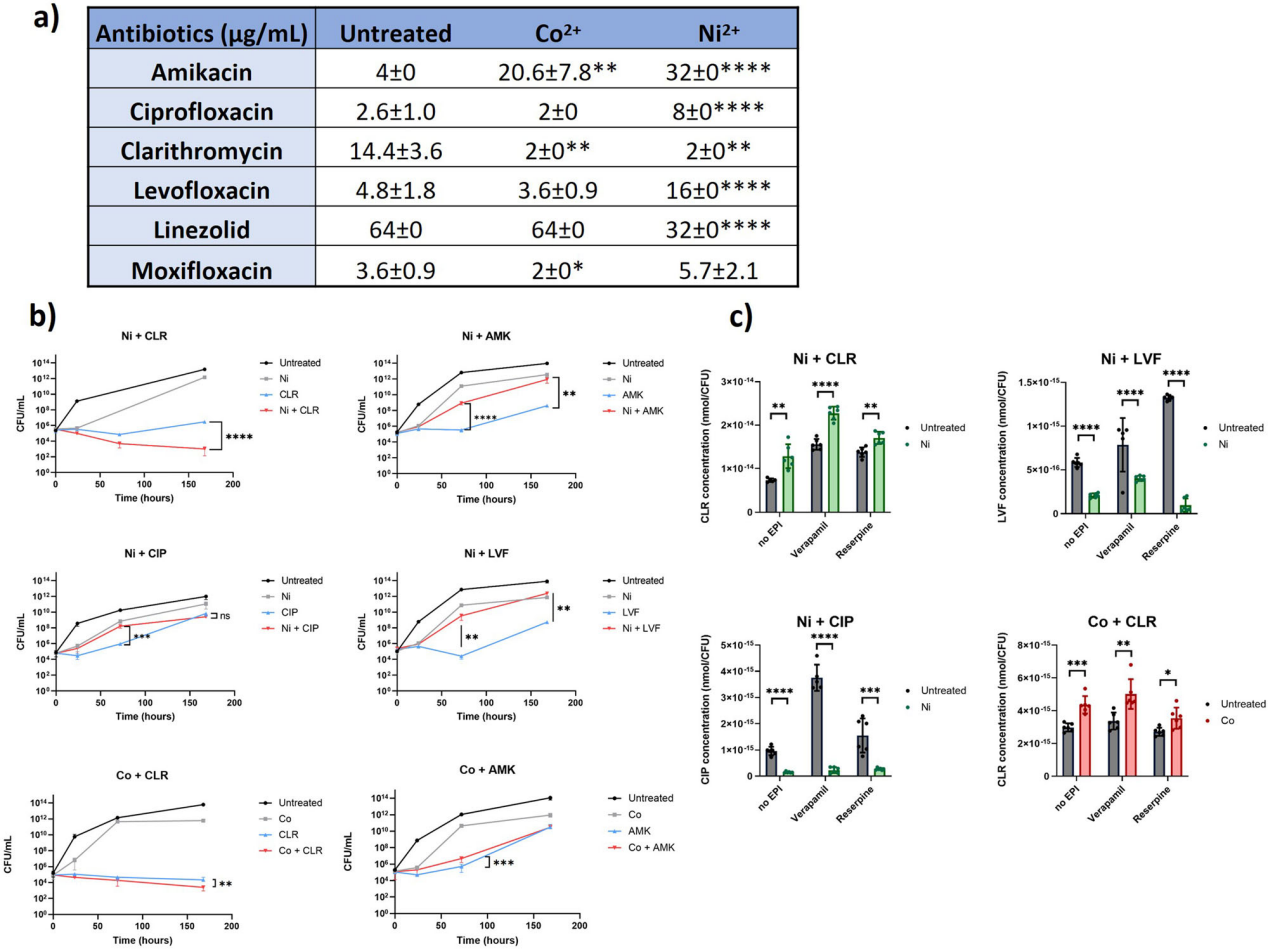
Transition metal treatment changed bacterial response to antibiotics. **a** MIC_50_ of *M. abscessus*, untreated or transition metal-treated, to clinically relevant antibiotics. Data are averaged from at least three biological replicates for each condition and expressed as mean ± standard deviation. Asterisks indicate levels of significance compared with untreated bacteria; the significance level of differences were determined by unpaired Student’s *t* tests, with *p < 0.05; **p < 0.01; ***p < 0.001; ****p < 0.0001. **b** Time-killing curves of *M. abscessus* with different ion-antibiotic combinations. The growth of bacterial cultures was monitored for 7 days (168 hours) and numbers of viable cells (CFU/mL) were quantified by plating on 7H10 agar. Figures are averaged from two biological replicates, each with three technical replicates. Error bars represent standard deviation for each time point. The significance level of differences between antibiotic (blue) and ion-antibiotic combinations (red) for each group were calculated using a one-way ANOVA test and marked as **p < 0.01; ***p < 0.001; ****p < 0.0001. **c** Intracellular accumulation of clarithromycin (CLR), ciprofloxacin (CIP) and levofloxacin (LVF) in *M. abscessus* in the presence of Ni^2+^ or Co^2+^. The efflux pump inhibitors verapamil and reserpine were used in combination with ions and antibiotics. The statistical significance levels of differences between the treated and untreated groups for each condition were calculated by unpaired *t* tests, with levels of significance indicated on top of each condition as *p < 0.05; **p < 0.01; ***p < 0.001; ****p < 0.0001. The data of each panel are averaged from two biological replicates, each with three technical replicates, and individual data points are shown for each replicate.

To investigate how transition metal ion treatments alter the bacterial response to antibiotics, REMA assays were performed to determine the MIC_50_ in the presence of sub-MIC_50_ of Co^2+^ or Ni^2+^ (Fig. 5a). The antibiotics selected for this assay belong to different classes: macrolide, aminoglycoside, fluoroquinolone and oxazolidinone, reflecting the complex treatment regimen for *M. abscessus* infections^42,43^.

Treatment with Co^2+^ produced a significant 5-fold increase of the MIC_50_ of amikacin, from 4 μg/mL for the untreated group to 20.6 μg/mL (*p* = 0.021). In contrast, reduced MIC_50_ values were observed on treatment with Co^2+^ for clarithromycin (from 14.4 μg/mL to 2 μg/mL, *p* = 0.004) and moxifloxacin (from 3.6 μg/mL to 2 μg/mL, *p* = 0.037). The MIC_50_ values for ciprofloxacin and levofloxacin, the other two fluoroquinolones similar to moxifloxacin, did not change with the Co^2+^ treatment. Similar changes for amikacin and clarithromycin were found for the Ni^2+^ treatments. Meanwhile, in contrast to the Co^2+^-treated groups, *M. abscessus* showed higher MIC_50_ for all three fluoroquinolones used in this study for the Ni^2+^ treatments, with a 3-fold increase in resistance to ciprofloxacin and levofloxacin (*p* = 0.0007 and *p* = 0.0004, respectively), and a statistically not significant 1.6-fold increase in resistance to moxifloxacin (*p* = 0.0647). Lastly, a Ni^2+^-specific change in bacterial response to linezolid was found, with a 2-fold decrease in MIC_50_ from 64 µg/mL to 32 µg/mL.

Based on the REMA results, six ion-antibiotic combinations that showed significant differences in MIC compared with untreated cultures were selected for a kinetic time-killing study: Ni^2+^ with clarithromycin (CLR), amikacin (AMK), ciprofloxacin (CIP), levofloxacin (LVF), as well as Co^2+^ with clarithromycin and amikacin (Fig. 5b). The kinetic experiments were carried out to further understand the effect of transition metal treatments on the drug tolerance profile of *M. abscessus*.

When treated with CLR at 1 × MIC_50_ concentration, the bacterial population was relatively stable over time, with a three-fold increase in CFU at the end of the assay (Fig. 5b). When CLR was supplemented with Ni^2+^, the bacterial population steadily decreased over time and the CFU of this culture was reduced by 3 log_10_ units in comparison to the CLR-only cultures after 7 days (*p* = 0.0001). This demonstrated a stronger bactericidal effect of the Ni^2+^-CLR combination compared with the CLR-only group. The opposite was observed for the combination of Ni^2+^ with AMK. When used alone, AMK maintained stable bacterial CFU during the first 72 hours and its effect stopped afterwards, leading to a bacterial growth of about 2 log_10_ units at t = 168 h. The combined treatment with Ni^2+^ and AMK yielded a growth curve that was initially intermediate between those of the Ni^2+^- and AMK-only groups, with the combined treatment showing bacterial CFU that were more than 2 log_10_ higher compared to the AMK-only group (*p* = 0.0001 at t = 72 h and *p* = 0.0044 at t = 168 h). At the end of the assay, the CFU for the combined treatment group was similar to that of the Ni^2+^-only group. The killing kinetics revealed somewhat similar trends for CIP and LVF. In particular, Ni^2+^ supplementation resulted in significantly higher bacterial growth after 72 hours of incubation than observed for the antibiotic-only groups (*p* = 0.0003 and *p* = 0.0073 for Ni^2+^+ CIP and Ni^2+^ + LVF groups, respectively). After 168 h, however, only the Ni^2+^ + LVF group still had more bacterial growth than the antibiotic-only treatment.

To summarise, the data of Fig. 5b demonstrate that Ni^2+^ treatments enhanced and reduced the killing efficacy of CLR and AMK, LVF, respectively, but had no significant impact on the efficacy of CIP. Similar to Ni^2+^, the combined Co^2+^-CLR treatment achieved a significant 1 log_10_ reduction in CFU compared to the CLR-only group after 7-day treatment (*p* = 0.0084). Meanwhile, the combined Co^2+^-AMK-treated bacteria showed a 9-fold higher CFU than the AMK-only group at T = 48 h (*p* = 0.0002) but this difference was no longer apparent at the end of assay (Fig. 5b).

### Transition metal treatment led to induction of *erm*(41) gene in *M. abscessus*

Induction of *erm(41)*, which encodes for an erythromycin ribosomal methylase that methylates the 23 s rRNA and prevents the action of macrolides, is most likely the major contributor to CLR resistance in *M. abscessus*^44^. It has been reported that *erm*(41) is induced under stress of antibiotic treatment. For example, 7-day incubation with a sub-lethal level of CLR was shown to induce the expression of *erm(41)*, increasing the MIC_50_ to this antibiotic^45^. In addition, upregulation of *erm(41)* was also observed for both the Co^2+^ and Ni^2+^ groups in our RNA-seq dataset, suggesting the link between transition metal stress and clarithromycin resistance. To validate these findings and investigate the involvement of *erm(41)* in the MIC_50_ changes to clarithromycin, *M. abscessus* was re-grown without CLR after 7-day exposure to 1 × MIC_50_ of CLR and the levels of *erm(41)* RNA were quantified for each condition. Consistent with previous literature, significant upregulation of this gene was found in CLR-treated bacteria (FC = 4.61 ± 1.19, *p* < 0.0001) compared with untreated bacteria. Similarly, a 7-day Co^2+^ incubation at a sub-MIC_50_ level led to an increase in the expression level of *erm*(41) (FC = 4.67 ± 0.90, *p* < 0.0001), while no *erm(41)* expression change was found in the Ni^2+^ group (FC = 1.08 ± 0.07, *p* = 0.0188). Combined treatments with Co^2+^ or Ni^2+^ and CLR also led to upregulation of *erm*(41) (Co^2+^ + CLR: 9.39 ± 0.27, Ni^2+^ + CLR: 6.02 ± 0.49, both *p* < 0.0001). Compared to the ion-only treatment groups, combined treatment with Co^2+^ or Ni^2+^ and CLR lead to significantly higher levels of *erm*(41) induction (both *p* < 0.0001). These results suggest that *M. abscessus* induced *erm*(41) under CLR and Co^2+^ exposure but not in treatment with Ni^2+^ alone. Therefore, changes in viable bacteria after the combined exposure to CLR and Ni^2+^ cannot be explained by changes in the expression of *erm(41)* alone. This indicates that another mechanism must be responsible for the synergistic effects of combined Ni^2+^ + CLR treatments on *M. abscessus* populations.

### Changes in antibiotic uptake are key to MIC differences following transition metal treatment

Multiple factors can affect bacterial response to antibiotics, and the data of this study indicate that, instead of *erm*(41) induction, an additional mechanism is present, which change the efficacy of CLR when the bacteria are also treated with Co^2+^ or Ni^2+^. Changes in the intracellular antibiotic concentrations can directly impact the efficacy of antibiotics and may contribute to multiple tolerance mechanisms of bacteria, such as the regulation of porin expression, remodelling of the cell envelope and expression of efflux pumps Sarathy et al.^46^. utilised techniques based on liquid chromatography-mass spectrometry to quantify antibiotic accumulation in *M. tuberculosis* for different growth conditions and antibiotics, including levofloxacin, rifampicin, ethambutol and linezolid^47^. Based on their approach, the accumulation of antibiotics in transition metal-treated *M. abscessus* was also quantified in this study, to understand the contribution of increased uptake to this susceptible phenotype (Fig. 5c).

When treated with Ni^2+^, the intracellular CLR abundance was 2-times higher than for untreated bacteria with *p* < 0.01. To determine if that increase was due to intracellular accumulation of CLR and not cell wall-associated binding in the presence of Ni^2+^, the same experiment was carried out with addition of efflux pumps inhibitors. When combined with either verapamil or reserpine, which are two efflux pump inhibitors, the intracellular abundances of the antibiotic increased for both groups, with Ni^2+^-treated bacteria showing a consistently higher accumulation of CLR compared to untreated bacteria. Similar to Ni^2+^-treated bacteria, those treated with Co^2+^ also showed increased uptake of CLR, in absence or in the presence of efflux pump inhibitors. A possible effect of Ni^2+^ on the uptake of ciprofloxacin and levofloxacin were also studied with the same methods. For both groups, the ion-treatment significantly reduced antibiotic accumulation inside the cells (Fig. 5c).

The results of the antibiotic uptake experiments correlate with the REMA assays, and this suggests that changes in the efficacies of the drugs on *M. abscessus* can be directly linked to changes in antibiotic uptake. Although the use of efflux pump inhibitors increased the intracellular concentrations of the antibiotics in both untreated and ion-treated bacteria, there were consistent differences between the former two groups. This indicates that changes in antibiotic uptake rather than efflux are the determinant of intracellular antibiotic accumulation in this assay.

## Discussion

The antimicrobial properties of transition metals have longstanding recognition, for example, the antibacterial, antifungal and antiviral roles of copper have inspired its use on surfaces^9^, and other metals such as silver and zinc have also been acknowledged^48^. Transition metal toxicity is achieved through multiple routes, including mis-metalation of proteins, inhibition of the uptake of other metals, interference in replication and transcription, and generation of reactive oxygen species (ROS)^7^. In response to metal toxicity, the metal resistance mechanisms utilised by bacteria are closely linked to the rise of AMR. For example, transition metals are involved in transcriptional regulation that could be linked to the expression of AMR-related genes, such as those encoding efflux pumps^19,49^. Additionally, transition metals can induce growth arrest, cell envelope remodelling and altered metabolism, leading to phenotypes with higher tolerance and persistence levels to certain antibiotics^11,31^. Therefore, transition metals can not only exert direct antimicrobial effects, but also potentially affect bacterial response to other antimicrobial agents. Understanding the involvement of transition metals in the development of AMR may provide important constraints on the diverse bacterial resistance machinery, which could inspire the development of new therapeutic strategies.

Environmental bacteria thrive in diverse conditions where they encounter different stresses, including high transition metal concentrations, such as in soil and wastewater. In the present study, *M. abscessus* was used as a model of environmental bacteria. More importantly, the ability of this opportunistic pathogen to infect humans and resist treatment with multiple antibiotic classes has made it a clinical nightmare. Further understanding of the AMR mechanisms of *M. abscessus*, and how these relate to its environmental niche, would help to identify new drug targets or drug combinations, and could lead to better strategies to prevent infections.

In this study, two transition metal ions were chosen based on their environmental abundances, toxicity, and feasibility of testing at the available laboratory conditions. The inhibitory concentrations of the metals to *M. abscessus* were determined by REMA, showing that *M. abscessus* is tolerant to both metals at sub-millimolar to millimolar levels, which are in alignment with those reported in previous literature^50^. Although our data did not support the direct use of metal ions as antimicrobials against *M. abscessus*, in this study, we further exploit the mechanisms of metal toxicity and bacterial response with a sub-inhibitory concentration of each metal. The actual experimental concentrations were chosen to exert significant stress on bacteria but still allow culture recovery for subsequent experiments. The chosen sub-MIC_50_ testing concentrations were further validated by dose-dependent growth curves, showing that the concentrations of Co^2+^ and Ni^2+^ fit the criteria.

This study has found that both Co^2+^ and Ni^2+^ treatments were associated with similar transcriptomic and metabolomic changes, including remodelling of the central carbon metabolism (Figs. 2 and 3). These findings may reflect the toxicology and general impact of transition metals on *M. abscessus*, but it is also possible that they are secondary effects of the similarly reduced growth rates that were observed for the Co^2+^ and Ni^2+^ groups. Under stress conditions such as nutrient deprivation, hypoxia and acidity, mycobacteria switch to a dormant state with reduced growth and physiological modifications for better adaptation and survival, including rewiring of the central carbon metabolism^51^. Meanwhile, activation of WhiB7 was identified for all ion-treated groups, and this represents a general stress response mechanism against metal toxicity (Supplementary Data 7). It was previously reported that WhiB7 responds to various other environmental stresses, such as heat shock, iron starvation and hypoxia^52–54^. As WhiB7 controls the expression of several AMR-conferring genes, including *eis*2 and *erm*(41), its induction under environmental stress may be directly linked to the rise of AMR to different antibiotics in *M. abscessus*.

The results of this study show that the general response of *M. abscessus* to sub-lethal levels of transition metals resembles changes that can be induced by slow growth and hence shares similarities with the responses to other stress conditions. Whilst the direct target of transition metal toxicity could not be identified in the current study due to the masking effects of bacterial dormancy, ion-specific responses were observed for Co^2+^ and Ni^2+^, reflecting their distinct chemistry and toxicity. In bioenergetic studies, OCR and ECAR were measured as indicators of respiration and the central carbon metabolism, respectively (Fig. 3a). Although the genes related to OXPHOS were downregulated for both the Co^2+^ and Ni^2+^ groups, there were no significant changes in the rate of respiration, as shown by a stable OCR. This may reflect the highly flexible respiratory chain utilised by mycobacteria. For example, alternative electron donors, such as alanine dehydrogenase and proline dehydrogenase, may be utilised aside from the conventional NADH dehydrogenase and succinate dehydrogenase, to maintain the menaquinone-menaquinol pool^55,56^. For the Co^2+^ group, there was no change in the NAD^+^/NADH ratio, which may indicate a normally functioning respiration chain (Fig. 3b). In addition, the activities of ETC components may be maintained through compensatory mechanisms, leading to stable activity of the pathway despite downregulation at the transcriptional level. This was observed for the succinate dehydrogenase complex, which acts as both complex II in the ETC and a component of the central carbon metabolism. Although downregulation of its subunits was identified in transcriptomic analyses, ^13^C flux measurements showed a stable turnover of the TCA metabolites, suggesting that its activity was maintained.

For the Ni^2+^-treated bacteria, a 12-fold higher NAD^+^/NADH ratio was determined despite stable OXPHOS activity (Fig. 3b). This suggests that the NAD^+^/NADH balance may be independent of OXPHOS activity, and that the main contribution may come from a TCA cycle deficiency, which rendered NAD^+^ conversion to NADH ineffective. Also specific to the Ni^2+^ group is the significantly reduced turnover of TCA cycle metabolites, such as 2-oxoglutarate. Following the ceased turnover of 2-oxoglutarate, the turnovers of succinate, fumarate and malate were found to be partly restored. This indicates that the bacteria are able to utilise alternative pathways, such as the glyoxylate shunt, to partly maintain their carbon catabolic activities. For example, it was previously demonstrated that the level of succinate was maintained during environmental stresses such as hypoxia, by activation of isocitrate lyase (ICL), to sustain OXPHOS activity and a healthy membrane potential^57^.

The current study shows that the activation of the glyoxylate shunt is a key mechanism employed by *M. abscessus* to defend against metal ion toxicity. The glyoxylate shunt represents an alternative carbon metabolic pathway, which is associated with the fatty acid metabolism in bacteria^58^. For both the Co^2+^ and Ni^2+^ groups of this study, metal ion treatments led to upregulation of ICL and reprogramming of the TCA cycle. This allows the bacteria to redirect the carbon flows and maintain functioning carbon metabolic pathways. Previous studies also identified upregulation of ICL and activation of the glyoxylate shunt in *M. abscessus* that encountered environmental stresses, such as nutrient deprivation and hypoxia, whilst they resided within biofilms and during macrophage infection^59,60^. The glyoxylate shunt was also linked to the broad antibiotic tolerance of *M. tuberculosis*, with the activation of ICL found after antibiotic treatment^61^. The glyoxylate shunt may therefore be an important contributor to the high AMR levels of *M. abscessus*. ICL and malate synthase have been studied as targets for the development of new treatment strategies for *M. tuberculosis*^62,63^, but reports on the effects of ICL inhibitors on *M. abscessus* are currently lacking. In this study, the time-killing properties of itaconate, a human immune metabolite and a known ICL inhibitor, were tested^41^. Combined treatments with Ni^2+^ and itaconate prevented bacterial regrowth during a 3-day incubation, leading to significant differences in viable cell counts compared to the itaconate-only group (Fig. 4). These results suggest possible synergistic effects between Ni^2+^ and itaconate, which should be further investigated.

Slower growth is a common mechanism of antibiotic tolerance, as this reduces the need for key metabolic pathways and the synthesis of new biomolecules. We may hence expect *M. abscessus* to show higher levels of resistance to antibiotic treatments as a combined result of metal ion toxicity and dormancy induced by slower growth. To test this hypothesis, the MIC_50_ to various clinically relevant antibiotics were tested on ion-treated and untreated bacteria (Fig. 5). These antibiotics span different classes and target multiple cellular pathways. In detail, amikacin, linezolid and clarithromycin are all protein synthesis-targeting antibiotics with different modes of action, while ciprofloxacin, levofloxacin and moxifloxacin are all fluoroquinolones that target DNA topoisomerase^64–66^. Overall, bacteria treated with either Co^2+^ or Ni^2+^ showed higher MIC_50_ to most of the antibiotics tested, and Ni^2+^ was able to induce wider changes in the MIC_50_ profiles than Co^2+^, and this may be a consequence of ion-specific effects.

In contrast to other antibiotics tested, the MIC_50_ for CLR was reduced significantly when supplemented with either Ni^2+^ or Co^2+^. Similarly, the time-killing curves of CLR showed improved antibiotic efficacy with faster killing when combined with either metal ion. This finding is of great interest, as CLR is recognised as a cornerstone of current treatment regimens for *M. abscessus*^42,67^. Induction of *erm*(41) is a major contributor to the high levels of CLR resistance in *M. abscessus*^68^. RNA-seq analyses identified upregulation of *erm(41)* genes in *M. abscessus* treated with Co^2+^ or Ni^2+^, and our qRT-PCR results confirm the induction of this gene after incubation with CLR combined with Co^2+^ or Ni^2+^. Despite such inductions in both ion-treated groups, we still observed a reduction in bacterial populations at the end of time-killing experiments. This indicates that even under the induction of the resistance-determining *erm(41)* gene, treatments with Co^2+^ or Ni^2+^ can enhance the efficacy of CLR through an additional mechanism, such as changes to the uptake of the antimicrobials.

Indeed, increased uptake of CLR was found to be the main factor that contributed to lower MIC values for *M. avium* and *M. smegmatis* in comparison to the parental drug erythromycin^69^, whereby changes in the intracellular concentrations of the former can directly explain the observed differences in MICs. We investigated the intracellular accumulation of CLR with an uptake assay and found that treatments with either Co^2+^ or Ni^2+^ could increase the intracellular concentration of the antibiotic (Fig. 5c). The use of efflux pump inhibitors in combination with each treatment group further confirmed that the effect of increased uptake can outcompete drug efflux, whereby the latter was previously reported to be related to CLR resistance in *M. abscessus*^70^. These results suggest that, despite the presence of a generally tolerant phenotype, increased CLR uptake may be the main factor for its increased efficacy against the bacteria, leading to lower MIC and higher susceptibility. The intracellular concentrations of LVF and CIP were also quantified and revealed a reduction in the Ni^2+^-treated groups, consistent with the higher MICs of these two antibiotics (Fig. 5c). Both fluoroquinolones and macrolides are hypothesised to passively diffuse across cell membranes due to their large sizes^69,71,72^. As the differential response in intracellular accumulation cannot be explained by general mechanisms, such as changes in membrane permeability, there is a significant need to better understand the details of antibiotic uptake and possible ion-drug interactions. For example, a previous study that investigated synthetic CLR complexes with transition metals, including Co^2+^ and Ni^2+^, found that the Co^2+^-CLR complex had a higher killing efficacy against *S. aureus*^73^, but the causal mechanisms responsible for the effect remains to be fully explored. It is possible, however, that bonding interactions between transition metal ions and CLR can enhance drug penetration, leading to higher potency.

Although this study did not show the direct killing properties of either Co^2+^ or Ni^2+^ on *M. abscessus* due to its high tolerance with MIC_50_ values at millimolar levels, the above presented data further shows the potential of using transition metals or transition metal-containing compounds to enhance bacterial killing in combination with other known antimicrobials^48,50^. Metallic nanoparticles such as Ag, Cu and Zn and their oxides have attracted interest as drug delivery systems, which allow target-specific delivery of antibiotics to minimise their adverse effects and enhance their efficacies, while release of metal ions from these nanoparticles could also exert antimicrobial effects on the targeted pathogen^74,75^. Metal nanoparticles have also been shown with activity against bacterial biofilms based on metal toxicity^76–79^. Understanding how *M. abscessus* respond to metal ions, and how could metal ion exposure lead to changes in its AMR levels could provide useful information and assist development of new therapeutics.

To conclude, this study utilised multiple techniques to characterise the antimicrobial mechanisms of transition metals on the environmental mycobacterium *M. abscessus*. Specifically, transition metals altered the transcriptome and bioenergetic profiles of *M. abscessus* and suppressed bacterial growth. *M. abscessus* was found to utilise a remodelled carbon metabolism with an induced glyoxylate shunt as a tolerance mechanism against stresses from Co^2+^ and Ni^2+^. In addition, transition metals also induce changes in MICs for several clinically important antibiotics, including CLR and AMK. Despite the overall tolerant phenotype, combined treatment of Co^2+^/Ni^2+^ with CLR led to enhanced killing effect bv promoting antibiotic uptake through a yet unknown mechanism. These findings warrant further investigation, considering the importance of CLR in the current treatment regimen of *M. abscessus* infections, and suggest the potential of using transition metals to improve the efficacy of currently using antibiotics against *M. abscessus*.

## Methods

### Culture conditions

*M. abscessus* ATCC19977 smooth morphotype was grown in a shaking incubator at 37 °C, 180 rpm in Middlebrook 7H9 media (Sigma) supplemented with 0.2% w/v glycerol, 0.2% w/v dextrose, 0.5% w/v bovine serum albumin (BSA), 0.08% w/v NaCl and 0.05% w/v tyloxapol. 7H10 agar plate (Sigma) was supplemented with 0.2% w/v glycerol, 0.2% w/v dextrose, 0.5% w/v BSA and 0.08% w/v NaCl for growth on solid media in a static 37 °C incubator. Bacteria were grown to mid-exponential phase (OD_600nm_ = 0.8-1) for use in different experiments, unless otherwise stated.

### Determination of MIC

REMA were performed according to Palomino et al.^80^. A decreasing concentration gradient of the drug to be tested was prepared by serial dilution of the corresponding stock in complete 7H9 medium in clear-bottomed 96-well plates (Greiner Bio-One). For measurements of MIC to antibiotics in the presence of ions, the complete 7H9 medium was supplemented with the ion to be tested at previously determined sub-MIC_50_ concentrations (Co^2+^, 0.625 mM; Ni^2+^, 2.5 mM) and used for plate loading. A decreasing concentration gradient of the drug to be tested was then prepared with a similar protocol. The *M. abscessus* culture was diluted in supplemented 7H9 medium to a density of approximately 5 ×10^5^ CFU/mL, and 100 µL of bacterial suspension was loaded into each well of the plate. The plates were incubated in a static incubator at 37 °C with 5% CO_2_ for 48 hours, after which 30 µL of 0.2% sterile resazurin solution (Sigma) was loaded into each well. The plates were further incubated for 24 hours for colour development. The extent of colour change was measured at an excitation wavelength λ = 535 nm and an emission wavelength λ = 590 nm with a Hidex Sense microplate reader (Hidex).

### Growth curves

Growth curve assays were performed in 125 mL conical flasks (Corning) with prewarmed complete 7H9, supplemented with transition metal ions at defined sub-MIC concentrations. Pre-cultures of bacteria were grown and used to inoculate these flasks at OD_600nm_ = 0.001. Inoculated flasks were incubated in a 37 °C shaking incubator and OD_600nm_ measurements were taken twice a day for three days to produce growth curves until the stationary phase.

Dose-dependent growth curves were performed in 30-mL square bottles (Thermal Fisher). Pre-cultures of *M. abscessus* were inoculated at OD_600nm_ = 0.001 into 10 mL of 7H9 media in each bottle. The medium in each bottle was supplemented with Co^2+^ or Ni^2+^ in a range of concentrations between 0. 125x and 2x MIC_50_. The bottles were then incubated in a shaking incubator at 37 °C. OD_600nm_ was measured daily to produce growth curves until the stationary phase was reached.

### Metallomic analyses with ICP-MS

Bacteria were grown to a final OD_600nm_ of ~1 in 250 mL Erlenmeyer flasks. For the ion-treatment groups, the complete 7H9 media were supplemented with inorganic ions at the following sub-MIC_50_ concentrations: Co^2+^, 0.625 mM; Ni^2+^, 2.5 mM. Untreated bacteria were grown in complete 7H9 media without ion supplementation as a control. Bacteria grown with and without the addition of Co^2+^ and Ni^2+^ were harvested by repeated centrifugation at 3000 × *g* for 10 minutes at room temperature, and the pellets were air-dried in a biological safety cabinet. The pellets were further prepared for analysis in the MAGIC Laboratories at Imperial College London and the inorganic ion concentrations were then determined at the Imaging and Analysis Centre of the Natural History Museum, using established methods^81,82^. Briefly, following microwave assisted digestion of the bacteria pellets with a 3 + 2 mixture of 15 M HNO_3_ and 30-32% H_2_O_2_ in a Milestone Ethos EZ oven fitted with an SK-10 high pressure rotor and 100 mL PFA vessels, the resulting solutions were dried on a hotplate in a laminar flow hood. The samples were then redissolved in 0.1 M HNO_3_ for analysis. The elemental concentrations were determined with a quadrupole ICP-MS (Agilent 7700x), operated in both ‘gas mode’ (with He in the reaction cell) and ‘no gas mode’ to optimise data quality for different elements. The concentration-certified reference materials ERM-BB184 (bovine muscle) and ERM-BB186 (pig kidney) from the Institute of Reference Materials and Measurement were analysed alongside the samples for quality control and results are typically in accord with reference values to within better than ±10%, with a few deviations up to about ±20‰, for elements where reference data are available (Na, Mg, K, Ca, Mn, Fe, Co, Cu, Zn, Cd). All labware, reagents and techniques for sample processing were optimised and monitored to ensure that results were not biased by extraneous ion contamination. Data was retrieved and analysed using Microsoft Excel. All results were normalised to the colony formation units and then expressed as enrichment (or depletion) factors relative to data obtained in control experiments with bacteria grown under the same conditions but without ion supplementation, to normalise for the effects of ions originally present in the 7H9 media.

### RNA extraction

Bacteria were grown in 25 mL flasks to reach OD_600nm_ ≈ 0.8. Sub-MIC concentrations of the transition metal ions were supplemented, and the flasks were further incubated at the same conditions for 2 hours. Bacteria were harvested by 10-minute centrifugation at 4000 × *g* at 4 °C and washed twice with cold sterile phosphate-buffered saline solution (PBS). Total RNA was extracted using a FastRNA Pro Blue kit (MP) according to the manufacturer’s instructions. The samples were treated with RNase-free Dnase (Promega) for 1 hour at 37 °C to remove contamination by genomic DNA. The samples were then purified with RNAeasy columns (Qiagen) according to the manufacturer’s instructions. The quantity and quality of RNA were assessed using a NanoDrop 1000 spectrophotometer (Thermo Fisher Scientific) and an Agilent 2100 Bioanalyzer with an RNA 6000 Nano LabChip kit (Agilent). All samples displayed a 260/280 ratio >2.0 and RNA integrity numbers >7.0. RNA sequencing was performed using an Illumina HiSeq4000 sequencer in the Imperial College BRC Genomics Facility. Samples were sequenced to obtain paired-end reads with a minimum of 75 bp in length.

### RNA sequencing

Data analysis was performed by Dr Nadia Fernandes, Imperial College London. The quality of the reads and the sequencing run were assessed using FastQC (https://www.bioinformatics.babraham.ac.uk/projects/fastqc/) and FastQ (https://www.bioinformatics.babraham.ac.uk/projects/fastq_screen/) screen. Reads were trimmed using fastp (https://academic.oup.com/bioinformatics/article/34/17/i884/5093234) and were aligned to the *Mycobacterium abscessus* genome (NCBI: ATCC19977/CU458896) (https://www.ncbi.nlm.nih.gov/nuccore/CU458896) using the MEM algorithm within the Burrows-Wheeler aligner (BWA) (http://bio-bwa.sourceforge.net/). Reads in the aligned BAM files were indexed and sorted using Samtools (http://www.htslib.org/) and reads per gene were counted using the Feature Counts tools from the Subread R package (http://subread.sourceforge.net/). Here the start and end of genes were determined using a GTF annotation file, also derived from NCBI, for the same *M. abscessus* genome. The dataset was normalised, and a PCA and differential gene analysis were performed using the recommended DESeq2 workflow (http://bioconductor.org/packages/devel/bioc/vignettes/DESeq2/inst/doc/DESeq2.html) to compare controls versus each treatment condition. Significantly differentially regulated genes were classified as genes that have an adjusted *p* value of <0.05 and fold change greater than 2. The KEGG database (https://www.genome.jp/kegg/pathway.html) was queried using the significantly differentially regulated genes, and the Cluster Profiler tool (https://bioconductor.org/packages/release/bioc/html/clusterProfiler.html) was used to predict possible pathways associated with these genes. GSEA was used for pathway analysis and provided an associated p-value with each of the predicted pathways, which was then used to surmise the most likely pathways affected by the treatment. Enzyme entries of the selected pathway of interest were obtained from KEGG Pathway database with *M. abscessus* genome annotations.

### Metabolite extraction with stable isotope (^13^C) labelling

All stable isotope-labelled chemicals were purchased from Cambridge Isotope Laboratories, UK. The ^13^C-labelled 7H9 media was prepared by substituting glycerol and dextrose in the supplement with [U-^13^C_6_] glucose and [U-^13^C_3_] glycerol at the same concentrations. *M. abscessus* cultures were harvested by 10-minute centrifugation at 4000 × *g* at 4 °C, and the pellets were washed once with cold sterile PBS. Pellets were resuspended in stable isotope-labelled 7H9 media and supplemented with Co^2+^ or Ni^2+^ at sub-MIC concentrations. Cultures were incubated for 2 hours in a 37 °C shaking incubator. After incubation, bacteria were harvested by centrifuging at 4000 × *g* at 4 °C for 10 minutes, washed once with cold sterile PBS, and resuspended in 1 mL of lysis solution (40% acetonitrile, 40% methanol, 20% double distilled (dd) H_2_O). Samples were kept on ice and 250 µL of 0.1 mm acid-washed zirconia beads were added to each tube. Bacteria were then lysed using a FastPrep-24 homogeniser (MP) twice at 6.0 m/s for 30 seconds, with a 5-minute interval on ice. Supernatants were transferred to 0.22 μm spin × column filters (Costar) and filtered by centrifuging at 15,000 × *g* for 30 minutes. After filtration, 100 µL of the flowthrough was mixed with 100 µL of acetonitrile supplemented with 0.2% acetic acid and centrifuged at 15,000 × *g* for 10 minutes. 100 µL of the mixture was loaded into polypropylene snap vials (Agilent Technologies) for LC-MS analysis. Protein concentrations in each filtered sample were quantified using a bicinchoninic acid assay (BCA) kit (Thermo Fisher) following the manufacturer’s instructions.

### Liquid chromatography-mass spectrometry (LC-MS)

Aqueous normal-phase liquid chromatography was performed with an Agilent 1290 Infinity II LC system with a binary pump, a temperature-controlled autosampler (set at 4 °C) and a temperature-controlled column compartment (set at 25 °C) with a Cogent Diamond Hydride Type C silica column (150 mm × 2.1 mm, with a dead volume of 315 µL). The elution of polar metabolites was performed using solvent A, consisting of deionised water (resistivity ~18 MΩ cm) with 0.2% acetic acid, and solvent B, consisting of acetonitrile with 0.2% acetic acid. The following gradient was applied at a flow rate of 0.4 mL/min: 0 minutes, 85% B; 0–2 minutes, 85% B; 3–5 minutes, 80% B; 6–7 minutes, 75% B; 8–9 minutes, 70% B; 10–11 minutes, 50% B; 11.1–14 minutes, 30% B; 14.1–25 minutes, 20% B; and 5-minutes of re-equilibration at 85% B. Accurate mass spectrometry was performed using an Agilent Accurate Mass 6545 QTOF mass spectrometer. Dynamic mass axis calibration was achieved by continuous infusion after the chromatography of a reference mass solution using an isocratic pump connected to an ESI ionisation source operated in positive-ion mode. The nozzle and fragmentor voltages were set to 2000 V and 100 V, respectively. The nebuliser pressure was set to 50 psig, and the nitrogen drying gas flow rate was set to 5 L/minute. The drying gas temperature was maintained at 300 °C. The MS acquisition rate was 1.5 spectra/sec, and mass-to-charge (m/z) data ranging from 50 to 1200 were stored. The instrument enabled accurate mass spectral measurements with an error of <5 parts per million (ppm), a mass resolution ranging from 10,000 to 45,000 over the m/z range of 121–955 atomic mass units, and a 100,000-fold dynamic range with picomolar sensitivity. The data were collected in centroid 4-GHz (extended dynamic range) mode. The detected m/z data were deemed to represent metabolites, which were identified based on unique accurate mass-retention times and MS/MS fragmentation identifiers for masses exhibiting the expected distribution of accompanying isotopomers. The typical variation in the abundance of most of the metabolites remained between 5 and 10% under these experimental conditions.

### LC-MS metabolomic data analysis

MassHunter Qualitative Analysis software was used to check the extracted ion chromatogram (EIC) for the m/z values of interest and obtain their retention times. MassHunter Profinder B8.0 and MassHunter Profiler Professional software were used in untargeted metabolomics to extract the features of metabolites, analyse with PCA and perform clustering analysis. MassHunter Profinder was also used to extract the stable isotope labelling patterns in targeted metabolomic experiments, from a list of metabolites of interest generated in MassHunter PCDL Manager. After shortlisting the m/z values of interest, online metabolite databases METLIN (https://metlin.scripps.edu) and ECMDB (https://ecmdb.ca) were used for metabolite annotation. The KEGG Pathway database (https://www.genome.jp/kegg/pathway.html) was referred to confirm the presence of annotated metabolites in *M. abscessus* and build the metabolic pathway in flux analyses.

### Bioenergetics analysis with Seahorse XFp analyser

An Agilent Seahorse XFp HS Mini extracellular flux analyser with XF 8-well cell culture microplates and cartridges (Agilent Technologies) was used for measurements of oxygen consumption rate (OCR) and ECAR. One day prior to the assay, the sensor cartridge was hydrated by soaking the calibrating plate with 200 µL of sterile ddH_2_O and prewarming it in a sealed bag in a static 37 °C incubator overnight. All 8 wells of the culture plate were coated with 50 µL of poly-D-lysine (PDL) overnight. The PDL was removed, and the plate was washed with sterile ddH_2_O and air-dried prior to bacteria addition. Two hours before the assay, the moisturised cartridge was loaded with 20 µL of either the ion solutions at 10 × sub-MIC_50_ concentrations or ddH_2_O for control wells, and then equilibrated with prewarmed calibrant in the calibrating plate in the static incubator for at least 2 hours, until the start of the assay. *M. abscessus* was grown to mid-exponential phase and cell density adjusted to OD_600nm_ = 0.51 with fresh pre-warmed complete 7H9. Aliquots of 1 mL were collected, and bacteria were harvested by 15,000 × *g* centrifugation for 5 min. The pellets were washed once and resuspended in unbuffered 7H9 media (pH = 7.4) with dextrose, glycerol, and NaCl supplemented at concentrations appropriate for the complete 7H9 medium. 90 µL of bacterial suspension was loaded onto each well of the PDL-coated sample plate, which was then centrifuged at 2500 × *g* for 10 minutes to settle bacteria that formed a monolayer at the bottom of the wells. The total volume of media in each well was topped up to 180 µL with unbuffered complete 7H9 media, and the plate was equilibrated at 37 °C until the start of the assay. The analyser was first calibrated with the sensor cartridge and calibrating plate. Immediately after calibration, the calibrating plate was replaced by a cell culture plate to start the measurement. The assay was set to run three cycles of 6-minute basal measurements, after which drugs were injected from sensor cartridge and mixed with the media. Measurements were taken for a further 30 cycles. The OCR and ECAR data were analysed and normalised with CFUs by the Seahorse Wave software. Average changes in OCR and ECAR from 3 replicate wells of one assay were plotted with Microsoft Excel and Prism.

### NAD^+^/NADH assay

Fresh reaction buffer was prepared according to San et al.^83^ and kept away from light prior to the assay. The buffer contains 0.2 M bicine (pH = 8, Sigma), 20% v/v ethanol, 8 mM ethylenediaminetetraacetic acid, 6.6 mM phenazine ethosulphate (PES, Sigma), and 0.84 mM thiazolyl blue tetrazolium bromide (Sigma). Nicotinamide adenine dinucleotides (NAD^+^ and NADH) standards (Sigma) were prepared in ddH_2_O. A fresh solution of alcohol dehydrogenase from *Saccharomyces cerevisiae* (Sigma) was prepared at 1 mg/mL (314 units/mL) in ddH_2_O prior to the assay. *M. abscessus* was grown to mid-exponential phase with or without the addition of sub-MIC concentrations of ions, and 1 mL culture aliquots were taken and centrifuged at 15,000 × *g* for 10 minutes to collect a pellet. Bacteria were resuspended in 250 µL of either 0.2 M HCl (for NAD^+^ samples) or 0.2 M NaOH (for NADH samples) and heated at 55 °C for 10 minutes. Samples were neutralised by the addition of 0.2 M NaOH (for NAD^+^ samples) or 0.2 M HCl (for NADH samples) and centrifuged at 15,000 rpm at 4 °C for 10 minutes. Supernatants were collected as samples for the assay. A clear-bottomed black 96-well plate (Greiner Bio-One) was loaded with 80 µL of the sample solutions. Standards of NAD^+^ and NADH were loaded in concentration gradients (NAD^+^: 0–2.5 µM; NADH: 0–0.1 µM). 100 µL of reaction buffer was added to each well and the plate was incubated in the dark at room temperature for 5 minutes. After incubation, 20 µL of alcohol dehydrogenase solution was loaded in each well and the plate was sent immediately to a plate reader for kinetic measurements at a wavelength of *λ* = 570 nm, taking place every 30 seconds over 10 minutes. Standard curves of NAD^+^ and NADH at different concentrations were produced by plotting changes in OD_570nm_ over time. Concentrations of NAD^+^ and NADH were determined separately in samples by comparing with standard curve gradients and the NAD^+^/NADH ratios were calculated for the ion-treated and untreated groups.

### Time-killing assay

Bacteria were grown to the mid-exponential phase and used to inoculate pre-warmed 125 mL Erlenmeyer flasks with complete 7H9 media and the supplemented drug combinations at OD_600nm_ = 0.005. The *t* = 0 time point was taken by plating on 7H10 sector plates for CFU counting and the flasks were incubated in a shaking incubator at 37 °C at 180 rpm. Further timepoints were taken at 1 day (*t* = 24 hours), 3 days (*t* = 72 hours), and 7 days (*t* = 168 hours) for counting of CFU. The CFU results were calculated and plotted in Microsoft Excel against time.

### Quantification of *erm(41)* expression by qRT-PCR

*M. abscessus* was grown in 7H9 media that were either untreated or treated with Co^2+^, Ni^2+^, clarithromycin, Co^2+^ plus clarithromycin, or Ni^2+^ plus clarithromycin at previously defined sub-MIC_50_ and MIC_50_ concentrations. The cultures were grown in a 37 °C shaking incubator for 7 days. After incubation, bacteria were harvested by centrifugation and washed with sterile PBS once to remove any residual ions or antibiotics. The bacterial pellets were resuspended in fresh 7H9 media without any treatment, and the cultures were incubated for 3 more days to allow bacterial recovery. After recovery, bacteria were harvested, and RNA was extracted and purified using the methods described in the previous section. cDNA was produced from each RNA sample using the reverse transcriptase kit (Qiagen) as per manufacturer’s instructions. qRT-PCR was performed to quantify the expression levels of *erm*(41) using the primers MAB_erm41_f (5’-CAG GGG AGT TCG TTG TGG AT-3’) and MAB_erm41_r (5’-CGG ACA TCT TCC TCG GCA AA = 3’). The expression levels of sigA (MAB_3009) were used as an internal control using the primers sigA_f (5’–ACAAGGCTTCAGGCGATTTC-3’) and sigA_r (5’-TTGCCGATCTGCTTGAGGTAG-3’). Data were retrieved and processed with Microsoft Excel. The ΔΔC_T_ values were calculated for *erm(41)* in each sample using untreated bacteria as the control, and the fold changes of the expression levels of *erm(41)* were calculated as 2^(- ΔΔC_T_)^84^.

### Quantification of antibiotic uptake

The intracellular uptake of antibiotics in the presence of ions and/or efflux pump inhibitors was measured with methods adapted from the studies of Sarathy and colleagues^47^. The efflux pump inhibitors verapamil and reserpine (Sigma) were prepared as 5 mM solutions in 90% DMSO. *M. abscessus* was collected from actively growing cultures by centrifugation at 3000 rpm at 4 °C and resuspended in complete 7H9 media with OD_600nm_ adjusted to 4. The ion to be tested was added at a sub-MIC_50_ concentration to the testing groups, while equal volumes of sterile water were added to the untreated control groups. Verapamil or reserpine were added immediately after ion supplementation at concentrations of 75 µM and 20 µM, respectively. The cultures were incubated for 3 minutes, then supplemented with 10 µM of the antibiotic to be tested and incubated for 2 hours at 37 °C. Additional tubes with the same bacterial cultures but without an ion or drug treatment were also prepared and used later as a biological matrix for the preparation of antibiotic standards. After incubation, cultures were immediately cooled on ice and 500 µL sample aliquots were collected. Bacteria were resuspended in an equal volume of cool sterile PBS, and triplicate 20 µL samples were taken from each tube for CFU plating on 7H10 sector agar plates. The bacteria were then washed once and centrifuged at 15,000 rpm at 4 °C for 15 minutes. The pellets were resuspended in 500 µL of a sterile glycine-HCl solution (pH = 3) and incubated in a shaking incubator at 37 °C overnight. The bacterial suspensions were cooled on ice after the overnight incubation, and 250 µL of 0.1 mm acid-washed zirconia beads (Sigma) were added to each tube. The cells were homogenised and filtered, and samples were prepared for LC-MS analysis, as described in a previous section. Gradients of the tested antibiotic were prepared by spiking the biological matrix at concentrations of 1–1000 nM and loaded using the same process. The samples were then analysed by LC-MS in positive-ion mode. The data was processed using Agilent Qualitative Analysis B07.00 software for EIC and retention time extraction, and a targeted metabolite list was created for the corresponding antibiotic. Agilent MassHunter Profinder 8.00 was used for feature extraction and further data processing was completed with Microsoft Excel. A standard curve for each antibiotic was then plotted with the ion count data and spiking concentrations, and these curves were then employed to determine the antibiotic concentrations of the samples. The data were normalised by CFU counts and expressed as nmol of antibiotic per CFU.

### Statistics

For all phenotypic experiments, at least three biological replicates and at least three technical replicates for each condition were set up and analysed. Data are presented as the mean values determined for replicates plus or minus standard deviation. Statistical tests were performed to determine and compare the significance levels of the results, and are indicated in each figure. Different levels of significance are indicated as asterisks based on *p* values. The tests were performed with Prism statistical analysis software. The statistical analysis for the LC-MS data was carried out using two-way ANOVA with Bonferroni multiple comparison tests, whereby *p* < 0.05 is considered significant.

## Supplementary information

Supplementary information

Supplementary data

## Data Availability

G.L.M. has full access to all the data generated in the study and takes responsibility for the integrity of the results and the accuracy of the data analysis. Data are available upon reasonable request. The RNA-Seq data are available in GEO under the accession number GSE243031.
