# Supplementary material for: Transition metal homoeostasis is key to metabolism and drug tolerance of Mycobacterium abscessus
Source: NPJ Antimicrob Resist. 2024 Sep 30;2:25. doi: 10.1038/s44259-024-00042-7 (PMC11442307; doi:10.1038/s44259-024-00042-7)
Supplement: Supplementary file 1 — Supplementary information [file 44259_2024_42_MOESM1_ESM.pdf]

Supplementary Figure 1. Dose-dependent growth curves of *M. abscessus* treated with different concentrations of Co^{2+} or Ni^{2+}.

Based on the MIC_{50} values determined by REMA assays, *M. abscessus* was grown with different concentrations of Co^{2+} or Ni^{2+} in concentrations ranging from 0.125x MIC_{50} to 2x MIC_{50}. Bacterial growth was monitored by OD_{600nm} measurement for 3 days. Data is averaged from 2 biological replicates, each with 2 technical replicates.

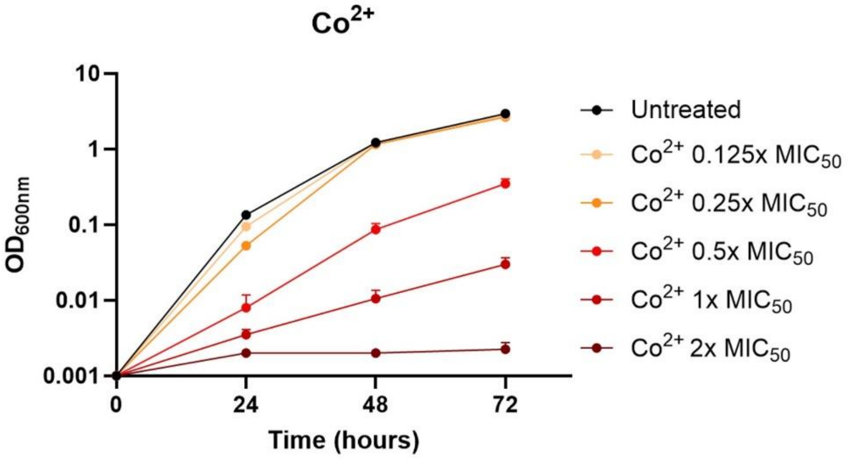

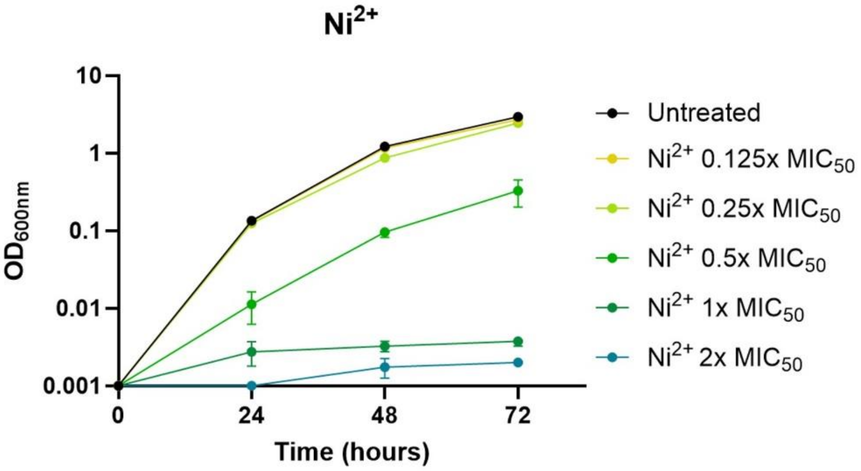

Supplementary Data

Supplementary Data 1. Changes in gene expression levels of *M. abscessus* after Co2+ treatment.

Supplementary Data 2. Changes in gene expression levels of *M. abscessus* after Ni2+ treatment.

Supplementary Data 3. Changes in the expression levels of genes in the TCA cycle and the glyoxylate shunt after transition metal ion treatment.

Supplementary Data 4. Changes in the expression levels of genes in glycolysis after transition metal ion treatment.

Supplementary Data 5. Changes in expression levels of genes related to biosynthesis of selected amino acids after transition metal ion treatment.

Supplementary Data 6. Changes in the expression levels of genes in oxidative phosphorylation (OXPHOS) after transition metal ion treatment.

Supplementary Data 7. Changes in the expression levels of genes in the WhiB7 regulon after transition metal ion treatment.
